# Prevalence and risk factors of anxiety and depression in patients with multi-drug/rifampicin-resistant tuberculosis

**DOI:** 10.3389/fpubh.2024.1372389

**Published:** 2024-03-27

**Authors:** Zhang Dan-ni, Zheng Guang-min, Du Yu-hua, Lin Ying, Wang Ting, Chen Yuan-yuan, Xie Yu-hong, Xiao Xin-cai

**Affiliations:** ^1^Department of Tuberculosis Control and Prevention, Guangzhou Chest Hospital, Guangzhou, China; ^2^Academy of Public Health, Guangzhou Medical University, Guangzhou, China; ^3^Department of Tuberculosis, Guangzhou Chest Hospital, Guangzhou, China

**Keywords:** anxiety, depression, multi-drug/rifampicin-resistant tuberculosis, prevalence, risk factors

## Abstract

**Background:**

Mental health disorders in patients with multi-drug or rifampicin-resistant tuberculosis (MDR/RR-TB) receive consistent attention. Anxiety and depression can manifest and may impact disease progression in patients with MDR/RR-TB. Given the heightened stressors resulting from the COVID-19 pandemic, this scenario is even more concerning.

**Objective:**

To evaluate the prevalence of and risk factors associated with anxiety and depression among patients with MDR/RR-TB in southern China.

**Methods:**

A facility-based cross-sectional study was undertaken at Guangzhou Chest Hospital in southern China, encompassing a cohort of 219 patients undergoing outpatient and inpatient treatment for MDR/RR-TB. Anxiety and depressive symptoms were assessed using the 7-Item Generalized Anxiety Disorder (GAD-7) scale and Patient Health Questionnaire-9 (PHQ-9). The ramifications of anxiety and depression were examined using univariate and multivariate logistic regression analyses, with odds ratios (ORs) and age- and sex-adjusted ORs (AORs) employed to quantify their influence. All data underwent statistical analysis using SPSS 25.0, with statistical significance established at *P* < 0.05.

**Results:**

Two hundred and nineteen individuals with MDR/RR-TB were included in the study. The prevalence of anxiety and depression was 57.53% (*n* = 126) and 65.75% (*n* = 144), respectively, with 33.3% (*n* = 73) of the participants experiencing both conditions simultaneously. Multivariate logistic regression analysis revealed that an age of 20–40 years [anxiety AOR = 3.021, 95% confidence interval (CI): 1.240–7.360; depression AOR = 3.538, 95% CI: 1.219–10.268], disease stigma (anxiety AOR = 10.613, 95% CI: 2.966–37.975; depression AOR = 4.514, 95% CI: 2.051–10.108) and poor physical health (anxiety AOR = 7.636, 95% CI: 2.938–19.844; depression AOR = 6.190, 95% CI: 2.468–15.529) were significant risk factors for moderate levels of anxiety and depression.

**Conclusions:**

We found that individuals with MDR/RR-TB had an elevated risk of anxiety and depression. To decrease the likelihood of unfavorable treatment outcomes, it is imperative to carefully monitor the psychological wellbeing of patients with MDR/RR-TB and promptly address any detrimental psychiatric conditions.

## Introduction

Different from ordinary fluctuations in mood and typical responses to daily life, individuals with anxiety disorders often experience intense and disproportionate levels of fear and worry. Such emotions are commonly accompanied by physical tension as well as other behavioral and cognitive symptoms (1). An estimated 4% of the global population currently grapples with an anxiety disorder, with approximately 301 million individuals worldwide affected in 2019, rendering anxiety disorders the most prevalent category among all mental health conditions (2).

Depression differs from regular mood changes and feelings about everyday life, as its episodes persist most of the day, nearly every day, for at least 2 weeks. Individuals experiencing depression may encounter disrupted sleep patterns and alterations in their appetite (3). Major depression represents a commonly occurring, serious, recurrent disorder associated with diminished role functioning, reduced quality of life, morbidity and mortality (4). As the most prevalent psychological illnesses in the population, anxiety and depression disorders commonly co-occur and considerably impact the physical and mental wellbeing of patients. The prevalence of depression and anxiety frequently correlates with physical health, and during periods of illness (5), adverse psychological states can hinder patients' engagement in health-related activities (6), such as medication adherence and treatment compliance. Conversely, illnesses can indirectly exacerbate patients' psychological conditions through factors such as stigma (7). In particular, individuals with infectious disorders often exhibit heightened rates of anxiety and depression (8).

Multi-drug/rifampicin-resistant tuberculosis (MDR/RR-TB) has emerged as a priority in tuberculosis prevention and control efforts (9). In 2020, an estimated 9.9 million individuals worldwide contracted tuberculosis, with 132,222 newly confirmed cases of MDR/RR-TB (10). A limited amount of research has been conducted on psychological disorders among patients with MDR/RR-TB. However, previous studies have reported varying incidences of anxiety, ranging from 6.8 to 54.6%, and depression, ranging from 7.8 to 55.9% (11–13). Patients undergoing treatment for MDR/RR-TB are susceptible to developing depression or anxiety symptoms due to the adverse effects of prolonged chemotherapy and anti-TB medications; thus, they face increased risks of poor treatment results, diminished quality of life and potential and avoidable injury or death by suicide (6). It is plausible that this percentage of psychological disorders may be even higher among individuals with MDR/RR-TB.

Although the prevalence of anxiety and depression in patients with MDR/RR-TB varies due to geographical and demographic differences, the potential harm to patients cannot be ignored, particularly given the COVID-19 pandemic. Measures such as physical distancing, isolation and other quarantine protocols imposed worldwide may have exacerbated the incidence of mental health issues (14). Initially, government-mandated interventions, including physical isolation, hindered patients' access to medical care and delayed their treatment. Moreover, patients were compelled to prolong their treatment regimen, diminishing their hope of recovery. Furthermore, patients with MDR/RR-TB are at heightened risk of exposure to and infection with COVID-19, leading to an increased likelihood of them developing psychiatric and mental health disorders. A previous study indicated a pooled prevalence of depression at 45% (95% CI: 37–54%) and anxiety at 47% (95% CI: 37–57%) among individuals with COVID-19 (15). Additionally, the financial burden of costly second-line anti-TB treatments coupled with temporary unemployment due to illness can lead to catastrophic expenditures and adverse psychological outcomes. A lack of awareness about emerging infectious illnesses and low self-health efficacy further exacerbates negative mood states (16). Hence, a re-assessment of the psychological challenges faced by patients with MDR/RR-TB is imperative in the context of the COVID-19 pandemic.

Guangdong Province, home to Guangzhou, boasts the largest economic output in China, and Guangzhou Chest Hospital receives a considerable influx of patients with drug-resistant TB from neighboring provinces, such as Hunan and Jiangxi (17). A considerable number of MDR/RR-TB cases are reported annually in Guangzhou. Interestingly, the majority of TB-afflicted residents in Guangzhou are immigrants, predominantly middle-aged and older adults, which are demographics more predisposed to experiencing anxiety and depression (18–20). However, there have been very few reports of anxiety and depression among patients with MDR/RR-TB. Hence, it is critical to comprehend the mental health of patients with MDR/RR-TB in Guangzhou. To provide a scientific foundation for the development of non-drug interventions and mental health strategies for patients with MDR/RR-TB, in this study, which is based on the representative research site of Guangzhou Chest Hospital, we aimed to evaluate the psychological status of patients with drug resistance during treatment, determine the incidence of anxiety and depression in these patients and investigate potential influencing factors.

## Materials and methods

### Population

Patients diagnosed with MDR/RR-TB and receiving both inpatient and outpatient care at Guangzhou Chest Hospital from February 2021 to August 2022 were enrolled in this single-center cross-sectional study. MDR/RR-TB was defined as infection with *Mycobacterium tuberculosis* demonstrating resistance to at least both isoniazid and rifampicin or rifampicin, as determined by *in vitro* drug susceptibility testing. Following the exclusion of 12 patients due to an inability to comprehend the study or audio–visual impairment, 233 patients met the study's inclusion criteria. An individual with audio–visual impairment refers to a person experiencing hearing or vision impairments and a comprehension deficit in language, including patients unable to understand and communicate despite repeated confirmation by the investigators. Written informed consent was obtained from all participants, with 14 individuals excluded for refusing to provide consent. The remaining 219 patients completed a survey, resulting in a response rate of 93.99%.

### Methods

**Figure 1:**
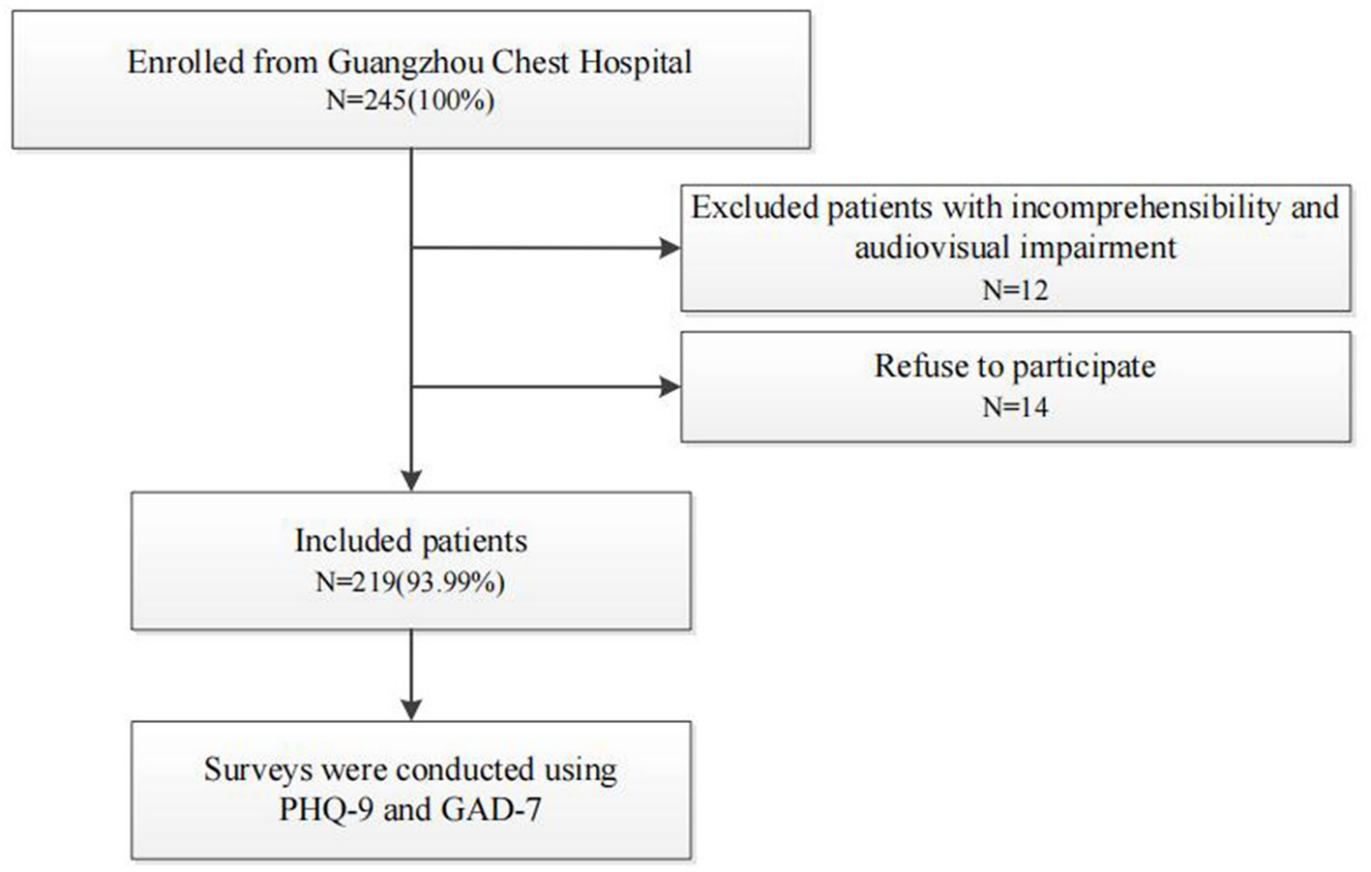
Flowchart of the study population. .

A structured questionnaire was used in this study, encompassing demographic baseline surveys pertaining to sex, age, educational level, marital status and residency status. To evaluate anxiety and depression, the 7-Item Generalized Anxiety Disorder (GAD-7) sale and Patient Health Questionnaire-9 (PHQ-9) were employed, both of which have demonstrated reliability in prior studies involving patients with TB, with good consistency (Cronbach's alpha values of 0.84 and 0.86 for the PHQ-9 scale and GAD-7, respectively) (21, 22). Patients possessing adequate comprehension and operational abilities were directed by investigators to scan a QR code using a mobile phone to complete the form, following explanations of the completion criteria. Patients with lower educational levels and limited autonomy underwent face-to-face questionnaire sessions, with each item explained and recorded with the respondents' consent. All investigators were proficient and experienced in conducting on-site investigations, ensuring minimal information input bias as the survey was conducted online, and all completed surveys were promptly exported to Microsoft Excel (https://office.microsoft.com/excel). The detailed procedural outline is depicted in Figure 1.

The GAD-7 scale, a screening scale developed by Spitzer et al. in 2006 (23), is commonly employed to evaluate participants' anxiety levels and diagnose generalized anxiety. It measures the intensity of immediate or recent feelings, such as tension, anxiety, fear, nervousness and related emotions. Each item on the GAD-7 scale is scored from 0 to 3 points, allowing for a maximum total score of 21 points. The cumulative score reflects the degree of anxiety experienced by the participant, with a score exceeding 5 points indicating the presence of anxiety.

Depressive symptoms experienced within the preceding 2 weeks were assessed using the PHQ-9. The patients rated the frequency of their depressive symptoms on a scale ranging from 0 to 3. The total score on the PHQ-9 ranges from 0 to 27, with a score of 10 or higher indicating clinically severe depressive symptoms (24). Scores from 1 to 4 indicate the absence of depression, whereas scores from 5 to 9 are indicative of mild depression. Moderate depression is denoted by scores of 10 to 14, whereas scores of 15 to 19 indicate moderate to severe depression. Finally, scores ranging from 20 to 27 are indicative of major depression.

### Statistical analysis

Data were extracted into a Microsoft Excel spreadsheet. Statistical analyses were conducted using SPSS 25.0 (IBM Corp., Armonk, NY, USA; released 2017). Interquartile ranges (IQRs) were used to describe the distribution of the GAD-7 and PHQ-9 scores among the interviewed patients. The Student's *t-*test was employed to analyze scores in instances where, following a normality test, a normal distribution was observed. Data not conforming to a normal distribution were examined using the Mann–Whitney *U*-test. Subsequently, scale scores were transformed into categorical variables, categorized as mild, moderate and above, to delineate variations in the prevalence of anxiety and depression symptoms across different attributes. Statistical evaluation of the prevalence differences was conducted using the chi-square test and Fisher's exact test using the statistical software SPSS 25.0. Univariate and multivariate logistic regression analyses were conducted to explore relevant elements and provide independent descriptions of the variables influencing anxiety and depression. Multivariate regression analysis included significant variables (*P* < 0.05) identified from the univariate analysis. Two regression models were constructed to identify the potential factors affecting the development of anxiety and depression. The first model comprehensively explored possible influencing factors, whereas the second model (*Model 1*) adjusted for age, gender and other variables, aiming to elucidate the degree of influence of other relevant aspects. The association between the research variables and anxiety and depression was evaluated using odds ratios (ORs), adjusted odds ratios (AORs) and 95% confidence intervals (95% CIs). *P* < 0.05 was considered statistically significant.

### Ethics

The study was approved by the Ethics Committee of the Guangzhou Chest Hospital. This project was funded by the Guangzhou Science and Technology Plan Project (No. 2023B03J1303). Except for 12 patients who declined to participate in the survey, all patients provided informed consent after being thoroughly briefed on the survey's objectives and procedures. They were assured that no personally identifiable information, such as names and ID numbers, would be disclosed. A dedicated ethics department tracked all of the collected data. There are no foreseeable ethical risks associated with this research.

## Results

### Characteristics

The study included 219 patients, with males constituting 62.6% (*n* = 137) and females 37.4% (*n* = 82). The patients were predominantly of Han Chinese ethnicity, comprising 95.4% (*n* = 209) of the total study population. The majority of patients (41.1%) were 20–40 years old, followed by 40–60 (37%) and over 60 years (18.3%). Farmers and unemployed individuals represented the primary occupations, accounting for 34.2 and 18.3%, respectively. The highest proportion of patients (63.5%; *n* = 139) had an annual income of < ¥30,000 (Table 1).

**Table 1:** Characteristics of patients with multi-drug or rifampicin-resistant tuberculosis.

**Characteristic**		***N***	**(%)**
Sex	Male	137	62.6%
	Female	82	37.4%
Age	≤ 20 y	8	3.7%
	20–40 y	90	41.1%
	40–60 y	81	37.0%
	≥60 y	40	18.3%
Nationality	Han	209	95.4%
	Other	10	4.6%
Educational level	Primary school or below	47	21.5%
	Middle school	67	30.6%
	High school	47	21.5%
	College	34	15.5%
	Bachelor's degree or above	24	11.0%
Marital status	Single	61	27.9%
	Married	146	66.7%
	Divorced	8	3.7%
	Widowed	4	1.8%
Occupational status	Unemployed	75	34.2%
	Farmer	40	18.3%
	Student	11	5.0%
	Retired	21	9.6%
	Cadres and workers	24	11.0%
	Service personnel	11	5.0%
	Other	37	16.9%
Registered residence address	Guangzhou	76	34.7%
	Outside Guangzhou	143	65.3%
Current address	Guangzhou	130	59.4%
	Outside Guangzhou	89	40.6%
Actual income/¥	< 30,000	139	63.5%
	30,000–50,000	46	21.0%
	50,000–100,000	26	11.9%
	>100,000	8	3.7%

### Prevalence and scores of anxiety and depression states in patients with MDR/RR-TB

**Figure 2:**
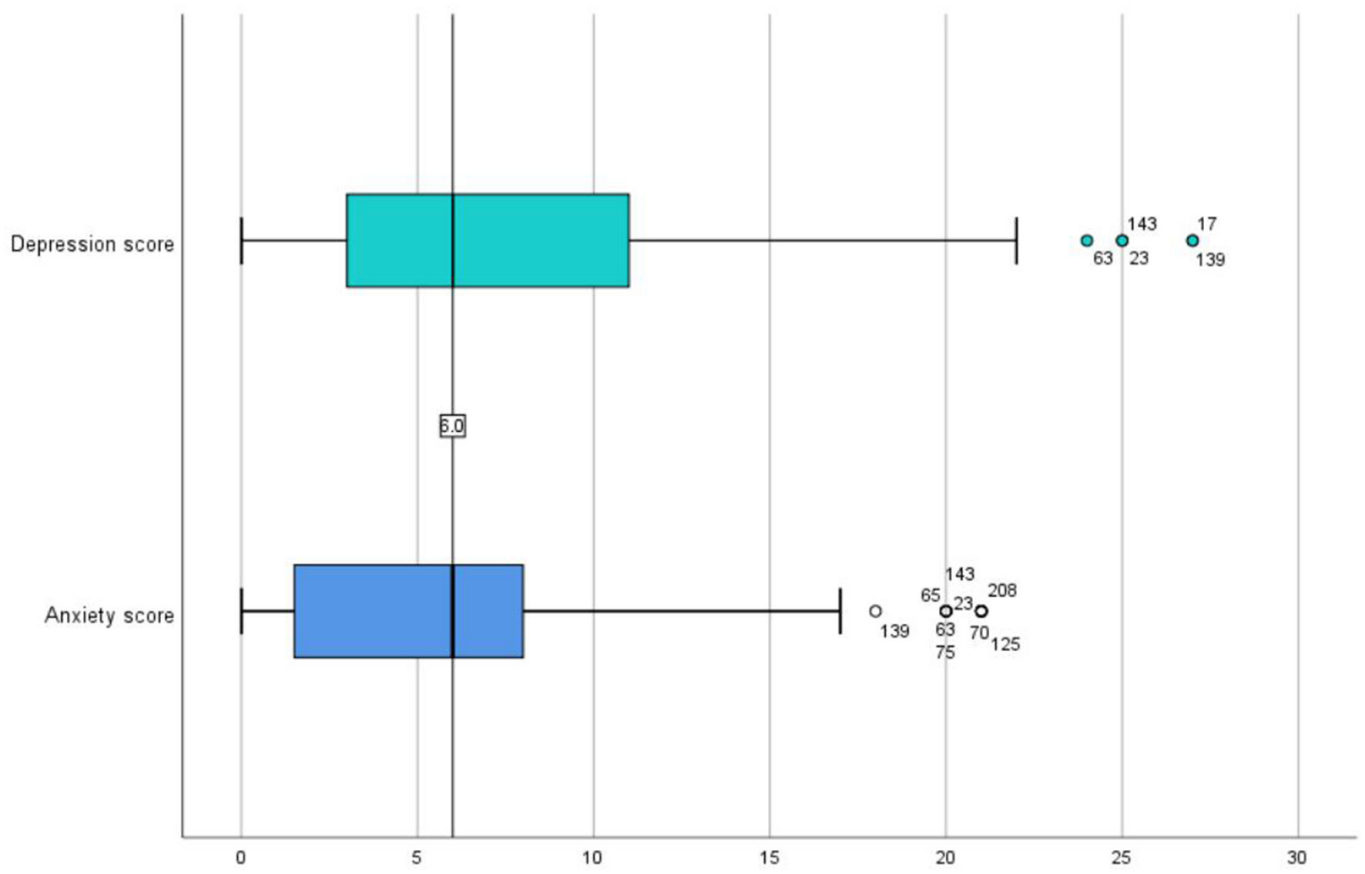
Overall anxiety and depression scores in patients with MDR/RR-TB.

In brief, 57.53% of patients reported experiencing anxious states, with a median GAD-7 score of 6 (IQR, 1–8). Mild (GAD-7 scores of 5–9) and moderate (GAD-7 score of 10) anxiety were found in 40.6 and 16.9% of the total population, respectively. Depressive states were reported by 65.75% of patients, with a median PHQ-9 score of 6. Mild (PHQ-9 scores of 5–9) and major (PHQ-9 score of 10) depression were found in 35.6 and 30.1% of the population, respectively (Tables 2, 4; Figure 2). Overall, depressive states were more prevalent than anxiety, at 57.53% compared to 65.75%. Of the study population, 28.3% (*n* = 63) did not exhibit either anxiety or depression, whereas 5.94% (*n* = 12) experienced anxiety alone, 14.2% (*n* = 31) experienced depression alone and 33.3% (*n* = 73) experienced both depression and anxiety.

**Table 2:** Overall anxiety and depression scores in patients with multi-drug or rifampicin-resistant tuberculosis.

	***N***	**Minimum**	**Maximum**	**Interquartile range**	**Median**	**Mean**	**Standard deviation**
Depression scores	219	0	27	3–11	6	7.64	5.876
Anxiety scores	219	0	21	1–8	6	5.84	5.062

We observed that women and individuals aged 20–40 years exhibited elevated rates of anxiety and depression. Our findings indicated that women scored higher than men for both anxiety and depression (*Z* = −3.846, *P* < 0.001; *Z* = −2.399, *P* = 0.016). Similarly, those aged 20–40 years demonstrated higher overall median scores (*H* = 10.121, *P* = 0.018; *H* = 8.177, *P* = 0.042) (Table 3).

**Table 3:** Hierarchical analysis of anxiety and depression scores in patients with multi-drug or rifampicin-resistant tuberculosis.

			***N***	**Interquartile range**	**Median**	**Mean**	**Standard deviation**	**Non-parametric tests**	***P***
Anxiety		Total	219	1.00–8.00	6.00	5.84	5.062		
	Sex	Male	137	1.00–7.00	4.00	4.90	4.726	*Z* = −3.846	< 0.001
		Female	82	4.00–9.00	7.00	7.40	5.228		
	Age	≤ 20 y	8	4.00–10.50	5.50	7.50	6.437	*H* = 10.121	0.018
		20–40 y	90	2.75–8.25	6.50	6.72	5.249		
		40–60 y	81	2.00–8.00	5.00	5.65	4.809		
		≥60 y	40	2.00–8.00	2.00	3.88	4.369		
Depression		Total	219	3.00–11.00	6.00	7.64	5.876		
	Sex	Male	137	3.00–10.00	6.00	6.93	5.604	*Z* = −2.399	0.016
		Female	82	4.00–12.25	8.00	8.82	6.161		
	Age	≤ 20 y	8	1.25–16.25	6.00	8.13	8.043	*H* = 8.177	0.042
		20–40 y	90	5.00–13.00	7.00	8.63	6.141		
		40–60 y	81	3.00–11.00	6.00	7.54	5.740		
		≥60 y	40	0.25–7.00	4.00	5.50	4.546		

### Univariate logistic regression analysis

According to the univariate logistic regression analysis, statistically significant differences (*P* < 0.05) were found in the anxiety levels of individuals based on their sex, age, actual income, perception of their ability to recover from illnesses, fear of illness, stigma, feelings of discrimination, physical condition and history of anxiety and depression (Table 4). Age, difficulty in healing, fear of illness, stigma, feeling discriminated against, physical condition and previous anxiety and depression all demonstrated statistically significant variations across different levels of depression (see Table 5).

**Table 4:** Univariate analysis related to anxiety and depression in patients with drug resistance.

**Characteristic**	**Anxiety**			χ^2^	***P***	**Depression**			χ^2^	***P***
	**None**	**Mild**	**Medium and above**			**None**	**Mild**	**Medium and above**		
	***n*** = **93, 42.5%**	***n*** = **89, 40.6%**	***n*** = **37, 16.9%**			***n*** = **75, 34.2%**	***n*** = **78, 35.6%**	***n*** = **66, 30.1%**		
Sex				15.918	< 0.001				4.082	0.130
Male	72 (77.42%)	47 (52.81%)	18 (48.65%)			52 (69.33%)	50 (64.1%)	35 (53.03%)		
Female	21 (22.58%)	42 (47.19%)	19 (51.35%)			23 (30.67%)	28 (35.9%)	31 (46.97%)		
Age				17.201	0.009				14.139	0.028
≤ 20 y	4 (4.3%)	2 (2.25%)	2 (5.41%)			4 (5.33%)	1 (1.28%)	3 (4.55%)		
20–40 y	26 (27.96%)	46 (51.69%)	18 (48.65%)			21 (28%)	39 (50%)	30 (45.45%)		
40–60 y	37 (39.78%)	30 (33.71%)	14 (37.84%)			29 (38.67%)	26 (33.33%)	26 (39.39%)		
≥60y	26 (27.96%)	11 (12.36%)	3 (8.11%)			21 (28%)	12 (15.38%)	7 (10.61%)		
Nationality				1.3	0.522				0.112	0.946
Han	90 (96.77%)	85 (95.51%)	34(91.89%)			72 (96%)	74 (94.87%)	63 (95.45%)		
Other	3 (3.23%)	4 (4.49%)	3 (8.11%)			3 (4%)	4 (5.13%)	3 (4.55%)		
Educational level				10.113	0.257				8.817	0.358
Primary school or below	19 (20.43%)	18 (20.22%)	10 (27.03%)			15 (20%)	16 (20.51%)	16 (24.24%)		
Middle school	33 (35.48%)	26 (29.21%)	8 (21.62%)			29 (38.67%)	18 (23.08%)	20 (30.3%)		
High school	22 (23.66%)	15 (16.85%)	10 (27.03%)			16 (21.33%)	16 (20.51%)	15 (22.73%)		
College	14 (15.05%)	16 (17.98%)	4 (10.81%)			10 (13.33%)	17 (21.79%)	7 (10.61%)		
Bachelor's degree or above	5 (5.38%)	14 (15.73%)	5 (13.51%)			5 (6.67%)	11 (14.1%)	8 (12.12%)		
Marital status				6.064	0.416				6.521	0.367
Single	23 (24.73%)	27 (30.34%)	11 (29.73%)			18 (24%)	23 (29.49%)	20 (30.3%)		
Married	67 (72.04%)	58 (65.17%)	21 (56.76%)			54 (72%)	52 (66.67%)	40 (60.61%)		
Divorced	2 (2.15%)	3 (3.37%)	3 (8.11%)			2 (2.67%)	3 (3.85%)	3 (4.55%)		
Widowed	1 (1.08%)	1 (1.12%)	2 (5.41%)			1 (1.33%)	0 (0%)	3 (4.55%)		
Occupational status				15.442	0.218				17.396	0.135
Unemployed	25 (26.88%)	34 (38.2%)	16 (43.24%)			25 (33.33%)	20 (25.64%)	30 (45.45%)		
Farmer	22 (23.66%)	15 (16.85%)	3 (8.11%)			17 (22.67%)	15 (19.23%)	8 (12.12%)		
Student	5 (5.38%)	4 (4.49%)	2 (5.41%)			5 (6.67%)	3 (3.85%)	3 (4.55%)		
Retired	14 (15.05%)	5 (5.62%)	2 (5.41%)			10 (13.33%)	6 (7.69%)	5 (7.58%)		
Cadres and workers	9 (9.68%)	9 (10.11%)	6 (16.22%)			7 (9.33%)	9 (11.54%)	8 (12.12%)		
Service personnel	6 (6.45%)	4 (4.49%)	1 (2.7%)			4 (5.33%)	6 (7.69%)	1 (1.52%)		
Other	12 (12.9%)	18 (20.22%)	7 (18.92%)			7 (9.33%)	19 (24.36%)	11 (16.67%)		
Registered residence address				3.299	0.192				0.121	0.941
Guangzhou city	38 (40.86%)	25 (28.09%)	13 (35.14%)			27 (36%)	26 (33.33%)	23 (34.85%)		
Outside Guangzhou	55 (59.14%)	64 (71.91%)	24 (64.86%)			48 (64%)	52 (66.67%)	43 (65.15%)		
Current address				0.610	0.737				0.502	0.778
Guangzhou	58 (62.37%)	51 (57.3%)	21 (56.76%)			45 (60%)	44 (56.41%)	41 (62.12%)		
Outside Guangzhou	35 (37.63%)	38 (42.7%)	16 (43.24%)			30 (40%)	34 (43.59%)	25 (37.88%)		
Treatment mode				2.659	0.265				2.178	0.337
Outpatient treatment	65 (69.89%)	70 (78.65%)	30 (81.08%)			52 (69.33%)	61 (78.21%)	52 (78.79%)		
Hospitalization	28 (30.11%)	19 (21.35%)	7 (18.92%)			23 (30.67%)	17 (21.79%)	14 (21.21%)		
Medical insurance reimbursement				0.082	0.960				0.636	0.727
Yes	62 (66.67%)	58 (65.17%)	25 (67.57%)			47 (62.67%)	53 (67.95%)	45 (68.18%)		
No	31 (33.33%)	31 (34.83%)	12 (32.43%)			28 (37.33%)	25 (32.05%)	21 (31.82%)		
Reimbursement proportion				7.149	0.307				9.213	0.162
Below 10%	40 (43.01%)	40 (44.94%)	20 (54.05%)			37 (49.33%)	34 (43.59%)	29 (43.94%)		
10–30%	17 (18.28%)	16 (17.98%)	6 (16.22%)			11 (14.67%)	18 (23.08%)	10 (15.15%)		
30–50%	8 (8.6%)	16 (17.98%)	5 (13.51%)			7 (9.33%)	7 (8.97%)	15 (22.73%)		
Above 50%	28 (30.11%)	17 (19.1%)	6 (16.22%)			20 (26.67%)	19 (24.36%)	12 (18.18%)		
Self-perceived income				2.303	0.680				6.041	0.196
Very low	54 (58.06%)	50 (56.18%)	25 (67.57%)			46 (61.33%)	41 (52.56%)	42 (63.64%)		
Insufficient	21 (22.58%)	24 (26.97%)	8 (21.62%)			13 (17.33%)	23 (29.49%)	17 (25.76%)		
Secondary	18 (19.35%)	15 (16.85%)	4 (10.81%)			16 (21.33%)	14 (17.95%)	7 (10.61%)		
Actual income/¥				13.197	0.040				10.483	0.106
< 30,000	64 (68.82%)	49 (55.06%)	26 (70.27%)			52 (69.33%)	41 (52.56%)	46 (69.7%)		
30,000–50,000	1 8(19.35%)	22 (24.72%)	6 (16.22%)			11 (14.67%)	21 (26.92%)	14 (21.21%)		
50,000–100,000	7 (7.53%)	17(19.1%)	2 (5.41%)			8 (10.67%)	14 (17.95%)	4 (6.06%)		
>100,000	4 (4.3%)	1(1.12%)	3 (8.11%)			4 (5.33%)	2 (2.56%)	2 (3.03%)		
Living situation				3.243	0.518				6.176	0.186
Live alone	16 (17.2%)	14(15.73%)	6 (16.22%)			9 (12%)	18 (23.08%)	9 (13.64%)		
Live with family	76 (81.72%)	70(78.65%)	30 (81.08%)			65 (86.67%)	56 (71.79%)	55 (83.33%)		
Live with others	1 (1.08%)	5(5.62%)	1 (2.7%)			1 (1.33%)	4 (5.13%)	2 (3.03%)		
Initial diagnosis or not				2.777	0.249				1.513	0.469
Yes (Initial treatment)	45 (48.39%)	54 (60.67%)	20 (54.05%)			39 (52%)	40 (51.28%)	40 (60.61%)		
No (retreatment)	48 (51.61%)	35 (39.33%)	17 (45.95%)			36 (48%)	38 (48.72%)	26 (39.39%)		
Time of diagnosis				1.287	0.864				5.045	0.283
< 1 month	9 (9.68%)	9 (10.11%)	3 (8.11%)			9 (12%)	9 (11.54%)	3 (4.55%)		
One month to half a year	27 (29.03%)	21 (23.6%)	8 (21.62%)			15 (20%)	23 (29.49%)	18 (27.27%)		
More than half a year	57 (61.29%)	59 (66.29%)	26 (70.27%)			51 (68%)	46 (58.97%)	45 (68.18%)		
Drug resistance status				0.596	0.742				0.357	0.837
One or two	43 (46.24%)	39 (43.82%)	19 (51.35%)			34 (45.33%)	38 (48.72%)	29 (43.94%)		
Three or more	50 (53.76%)	50 (56.18%)	18 (48.65%)			41 (54.67%)	40 (51.28%)	37 (56.06%)		
Drug resistance category				1.129	0.890				2.260	0.688
Only for the first line	66 (70.97%)	65 (73.03%)	25 (67.57%)			57 (76%)	55 (70.51%)	44 (66.67%)		
Only for the second line	5 (5.38%)	4 (4.49%)	1 (2.7%)			4(5.33%)	3 (3.85%)	3 (4.55%)		
For the first and second lines	22 (23.66%)	20 (22.47%)	11 (29.73%)			14(18.67%)	20 (25.64%)	19 (28.79%)		
Feel incurable				53.473	< 0.001				39.320	< 0.001
Never	31 (33.33%)	11 (12.36%)	2 (5.41%)			28(37.33%)	12 (15.38%)	4 (6.06%)		
Sometimes	46 (49.46%)	53 (59.55%)	11 (29.73%)			35(46.67%)	44 (56.41%)	31 (46.97%)		
Often	14 (15.05%)	23 (25.84%)	11 (29.73%)			10(13.33%)	20 (25.64%)	18 (27.27%)		
Almost every day	2 (2.15%)	2 (2.25%)	13 (35.14%)			2(2.67%)	2 (2.56%)	13 (19.7%)		
Fear of illness				81.616	< 0.001				56.626	< 0.001
Never	51 (54.84%)	13 (14.61%)	2 (5.41%)			42(56%)	16 (20.51%)	8 (12.12%)		
Sometimes	36 (38.71%)	55 (61.8%)	12 (32.43%)			27(36%)	45 (57.69%)	31 (46.97%)		
Often	5 (5.38%)	20 (22.47%)	15 (40.54%)			5 (6.67%)	17 (21.79%)	18 (27.27%)		
Almost every day	1 (1.08%)	1 (1.12%)	8 (21.62%)			1 (1.33%)	0 (0%)	9 (13.64%)		
Feel embarrassed				25.253	< 0.001				18.214	< 0.001
Yes	48 (51.61%)	67 (75.28%)	34 (91.89%)			38 (50.67%)	56 (71.79%)	55 (83.33%)		
No	45 (48.39%)	22 (24.72%)	3 (8.11%)			37 (49.33%)	22 (28.21%)	11 (16.67%)		
Feel discriminated against and evaded				31.509	< 0.001				25.528	< 0.001
Never	50 (53.76%)	19 (21.35%)	9 (24.32%)			40 (53.33%)	20 (25.64%)	18 (27.27%)		
Sometimes	34 (36.56%)	55 (61.8%)	15 (40.54%)			30 (40%)	45 (57.69%)	29 (43.94%)		
Often	7 (7.53%)	13 (14.61%)	9 (24.32%)			5 (6.67%)	10 (12.82%)	14 (21.21%)		
Almost every day	2 (2.15%)	2 (2.25%)	4 (10.81%)			0 (0%)	3 (3.85%)	5 (7.58%)		
Physical condition				24.323	< 0.001				26.600	< 0.001
Inferior	13 (13.98%)	19 (21.35%)	18 (48.65%)			9 (12%)	15 (19.23%)	26 (39.39%)		
Commonly	66 (70.97%)	67 (75.28%)	18 (48.65%)			55 (73.33%)	56 (71.79%)	40 (60.61%)		
Very good	14 (15.05%)	3 (3.37%)	1 (2.7%)			11 (14.67%)	7 (8.97%)	0 (0%)		
Frequency of sports activities				1.882	0.758				2.344	0.673
Nothing	33 (35.48%)	33 (37.08%)	16 (43.24%)			26 (34.67%)	27 (34.62%)	29 (43.94%)		
Once a week	24 (25.81%)	26 (29.21%)	7 (18.92%)			22 (29.33%)	19 (24.36%)	16 (24.24%)		
More than once a week	36 (38.71%)	30 (33.71%)	14 (37.84%)			27 (36%)	32 (41.03%)	21 (31.82%)		
Have you ever had anxiety or depression				9.374	0.009				10.625	0.005
No	89 (95.7%)	77 (86.52%)	29 (78.38%)			73 (97.33%)	68 (87.18%)	54 (81.82%)		
Yes	4 (4.3%)	12 (13.48%)	8 (21.62%)			2 (2.67%)	10 (12.82%)	12 (18.18%)		
Are there chronic diseases or comorbidities				0.864	0.649				1.268	0.530
Yes	61 (65.59%)	64 (71.91%)	25 (67.57%)			48 (64%)	54 (69.23%)	48 (72.73%)		
No	32 (34.41%)	25 (28.09%)	12 (32.43%)			27 (36%)	24 (30.77%)	18 (27.27%)		

**Table 5:** Multivariate logistic regression analysis results of anxiety in different groups.

**Anxiety Scale**	**Characteristic**	**Group**				**Model 1**		
			***P***	**OR**	**95% CI**	***P***	**AOR**	**95% CI**
Mild	Sex	Male	0.003	0.269	0.112–0.644	0.007	0.389	0.196–0.773
		Female	Reference					
	Age	≤ 20 y	0.200	0.216	0.021–2.251	0.669	0.659	0.097–4.465
		20–40 y	0.250	2.006	0.612–6.576	0.015	3.021	1.240–7.360
		40–60 y	0.875	1.091	0.367–3.240	0.234	1.694	0.711–4.036
		≥60 y	Reference					
	Actual income	<30,000	0.192	5.275	0.433–64.266	0.138	5.683	0.571–56.600
		30,000–50,000	0.062	11.583	0.881–152.336	0.064	9.242	0.876–97.495
		50,000–100,000	0.039	17.099	1.154–253.431	0.023	16.856	1.469–193.459
		>100,000	Reference					
	Treatment mode	Outpatient treatment	0.027	2.800	1.128–6.953	0.033	2.248	1.069–4.727
		Hospitalization	Reference					
	Time of diagnosis	<1 month	0.522	1.577	0.391–6.361	0.728	0.827	0.283–2.414
		1 month to half a year	0.316	0.619	0.243–1.580	0.074	0.506	0.240–1.068
		More than half a year	Reference					
	Feel incurable	Never	0.279	0.489	0.134–1.787	0.002	0.218	0.082–0.578
		Sometimes	0.236	0.543	0.197–1.492	0.209	0.606	0.278–1.323
		Often and above	Reference					
	Fear of illness	Never	0.003	0.136	0.036–0.514	<0.001	0.078	0.025–0.241
		Sometimes	0.218	0.469	0.141–1.563	0.103	0.426	0.153–1.187
		Often and above	Reference					
	Feel embarrassed	Yes	0.048	2.364	1.006–5.554	0.002	2.879	1.472–5.628
		No	Reference					
	Feel discriminated against and evaded	Never	0.581	0.686	0.181–2.608	0.004	0.219	0.079–0.612
		Sometimes	0.287	1.970	0.566–6.864	0.903	0.941	0.355–2.494
		Often and above	Reference					
	Physical condition	Inferior	0.177	1.973	0.736–5.293	0.097	2.027	0.880–4.669
		Average and very good	Reference					
	Have you ever had anxiety or depression	No	0.314	0.478	0.113–2.011	0.096	0.359	0.107–1.198
		Yes	Reference					
Medium and above	Sex	Male	0.021	0.232	0.067–0.803	0.015	0.346	0.146–0.816
		Female	Reference					
	Age	≤ 20 y	0.705	0.527	0.019–14.487	0.466	2.240	0.256–19.605
		20–40 y	0.262	3.072	0.433–21.806	0.044	4.132	1.039–16.428
		40–60 y	0.318	2.574	0.403–16.445	0.132	2.839	0.730–11.045
		≥60 y	Reference					
	Actual income	<30,000	0.694	0.638	0.068–5.973	0.930	0.927	0.111–5.249
		30,000–50,000	0.852	0.794	0.070–9.033	0.814	0.801	1.195–7.600
		50,000–100,000	0.653	0.514	0.028–9.341	0.695	0.637	0.671–3.951
		>100,000	Reference					
	Treatment mode	Outpatient treatment	0.006	7.467	1.776–31.402	0.048	2.747	1.010–7.471
		Hospitalization	Reference					
	Time of diagnosis	<1 month	0.212	3.750	0.471–29.861	0.469	0.583	0.136–2.511
		1 month to half a year	0.786	1.216	0.296–4.984	0.105	0.442	0.165–1.185
		More than half a year	Reference					
	Feel incurable	Never	0.229	0.286	0.037–2.196	<0.001	0.042	0.008–0.209
		Sometimes	0.004	0.143	0.038–0.531	<0.001	0.126	0.048–0.331
		Often and above	Reference					
	Fear of illness	Never	<0.001	0.023	0.003–0.192	<0.001	0.012	0.002–0.064
		Sometimes	0.003	0.113	0.027–0.480	<0.001	0.087	0.028–0.271
		Often and above	Reference					
	Feel embarrassed	Yes	0.010	8.685	1.692–44.581	<0.001	10.613	2.966–37.975
		No	Reference					
	Feel discriminated and evaded	Never	0.734	1.355	0.235–7.816	<0.001	0.115	0.036–0.372
		Sometimes	0.357	2.153	0.421–11.003	0.018	0.261	0.086–0.793
		Often and above	Reference					
	Physical condition	Inferior	0.004	6.797	1.874–24.646	<0.001	7.636	2.938–19.844
		Average and very good	Reference					
	Have you ever had anxiety or depression	No	0.089	0.225	0.040–1.258	0.014	0.191	0.052–0.711
		Yes	Reference					

Model 1 was adjusted for confounding factors, such as sex and age.

### Multivariate logistic regression analysis

In addition to variables that exhibited significant variations after univariate analysis, we incorporated factors such as treatment mode, time to diagnosis and drug resistance status into the multivariate analysis to ensure comprehensive coverage of potentially significant factors. We combined the options of feeling incurable, fear of illness, and feeling discriminated against and left out since there were few or no individuals. Given the initial dissimilar distribution of age and sex, we established a regression model adjusted for both sex and age (*Model 1*) to estimate the AOR for each variable.

The multivariate logistic regression analysis unveiled disease stigma (i.e., feeling ashamed) as an independent risk factor for moderate and high levels of anxiety (OR = 2.879, 95% CI: 1.472–5.628). Upon adjusting for confounding variables, the risk level increased by 10.613-fold (95% CI: 2.966–37.975). Furthermore, anxiety levels were notably elevated in patients who perceived their physical state negatively (moderate and above, AOR = 7.636, 95% CI: 2.938–19.844). Following adjustment, the anxiety risk remained higher in the 20–40 years age group than in the other age groups (mild anxiety AOR = 3.021, 95% CI: 1.240–7.360; moderate anxiety AOR = 4.132, 95% CI: 1.240–7.360). An income ranging from ¥50,000 to 100,000 emerged as a risk factor for moderate anxiety relative to incomes exceeding ¥100,000 (AOR = 16.856, 95% CI: 1.469–193.459). Notably, patients with MDR/RR-TB receiving outpatient care faced heightened anxiety risks (mild anxiety: AOR = 2.248, 95% CI: 1.069–4.727; moderate anxiety: AOR = 2.747, 95% CI: 1.010–7.471) (Table 5).

Stigma (i.e., feeling ashamed) emerged as an independent risk factor for depression, and its impact was further compounded by the severity of depression (AOR = 4.514, 95% CI: 2.051–10.108). Upon controlling for confounding variables, the adverse effect of a negative physical status assessment on moderate and higher levels of depression became more pronounced (OR = 3.615, 95% CI: 1.259–10.383; AOR = 6.190, 95% CI: 2.468–15.529). Notably, individuals aged 20–40 years faced the highest risk for depressive states (mild AOR = 3.247, 95% CI: 1.282–8.220; moderate and above AOR = 3.538, 95% CI: 1.219–10.268). Protective factors included feeling curable (mild depression: AOR = 0.231, 95% CI: 0.085–0.627; moderate depression: AOR = 0.057, 95% CI: 0.016–0.201), never fearing illness (mild depression: AOR = 0.132, 95% CI: 0.042–0.414; moderate depression: AOR = 0.047, 95% CI: 0.014–0.155), never experiencing avoidance or discrimination (mild depression: AOR = 0.211, 95% CI: 0.065–0.686; moderate depression: AOR = 0.123, 95% CI: 0.039–0.392) and having no prior history of anxiety or depression (moderate depression: AOR = 0.145, 95% CI: 0.031–0.688) (Table 6).

**Table 6:** Multivariate logistic regression analysis results of depression in different groups.

**Depression scale**	**Characteristic group**					**Model 1**		
			***P***	**OR**	**95% CI**	***P***	**AOR**	**95% CI**
Mild	Age	≤ 20 y	0.279	0.246	0.019–3.315	0.489	0.437	0.042–4.561
		20–40 y	0.113	2.384	0.814–6.980	0.013	3.247	1.282–8.220
		40–60 y	0.828	1.120	0.402–3.123	0.322	1.568	0.644–3.822
		≥60 y	Reference					
	Treatment mode	Outpatient treatment	0.190	1.785	0.750–4.247	0.128	0.185	0.843–3.865
		Hospitalization	Reference					
	Drug resistance status	One or two	0.019	2.965	1.193–7.365	0.580	1.202	0.626–2.309
		Three or more	Reference					
	Drug resistance category	Only for the first line	0.036	0.320	0.110–0.931	0.201	0.593	0.266–1.321
		Only for the second line	0.417	0.425	0.054–3.354	0.397	0.477	0.086–2.647
		For the first and second lines	Reference					
	Feel incurable	Never	0.266	0.504	0.151–1.687	0.004	0.231	0.085–0.627
		Sometimes	0.516	0.722	0.269–1.933	0.308	0.642	0.274–1.505
		Often and above	Reference					
	Fear of illness	Never	0.019	0.209	0.056–0.777	0.001	0.132	0.042–0.414
		Sometimes	0.639	0.754	0.232–2.451	0.283	0.556	0.190–1.623
		Often and above	Reference					
	Feel embarrassed	Yes	0.459	1.363	0.601–3.095	0.015	2.347	1.179–4.670
		No	Reference					
	Feel discriminated against and evaded	Never	0.274	0.462	0.116–1.844	0.010	0.211	0.065–0.686
		Sometimes	0.651	0.735	0.193–2.794	0.346	0.574	0.181–1.821
		Often and above	Reference					
	Physical condition	Inferior	0.566	1.348	0.486–3.743	0.102	2.190	0.857–5.600
		Average and very good	Reference					
	Have you ever had anxiety or depression	No	0.070	0.197	0.034–1.139	0.068	0.232	0.048–1.116
		Yes	Reference					
Medium and above	Age	≤ 20 y	0.727	1.500	0.154–14.592	0.568	1.684	0.281–10.097
		20–40 y	0.041	3.994	1.055–15.117	0.020	3.538	1.219–10.268
		40–60 y	0.214	2.238	0.629–7.968	0.077	2.498	0.906–6.891
		≥60 y	Reference					
	Treatment mode	Outpatient treatment	0.138	2.096	0.788–5.578	0.080	2.052	0.917–4.588
		Hospitalization						
	Drug resistance status	One or two	0.028	3.180	1.136–8.903	0.991	0.996	0.503–1.973
		For three or more	Reference					
	Drug resistance category	Only for the first line	0.035	0.285	0.089–0.914	0.110	0.512	0.225–1.163
		Only for the second line	0.571	0.514	0.051–5.143	0.471	0.530	0.095–2.971
		For the first and second lines	Reference					
	Feel incurable	Never	0.026	0.180	0.040–0.812	< 0.001	0.057	0.016–0.201
		Sometimes	0.129	0.454	0.164–1.259	0.007	0.311	0.133–0.725
		Often and above	Reference					
	Fear of illness	Never	0.004	0.124	0.030–0.513	< 0.001	0.047	0.014–0.155
		Sometimes	0.306	0.538	0.164–1.763	0.010	0.253	0.089–0.718
		Often and above	Reference					
	Feel embarrassed	Yes	0.081	2.420	0.898–6.523	< 0.001	4.514	2.051–10.108
		No	Reference					
	Feel discriminated against and evaded	Never	0.644	0.710	0.166–3.035	< 0.001	0.123	0.039–0.392
		Sometimes	0.462	0.591	0.146–2.397	0.012	0.227	0.072–0.717
		Often and above	Reference					
	Physical condition	Inferior	0.017	3.615	1.259–10.383	< 0.001	6.190	2.468–15.529
		Average and very good	Reference					
	Have you ever had anxiety or depression	No	0.028	0.132	0.022–0.804	0.015	0.145	0.031–0.688
		Yes	Reference					

Model 1 was adjusted for confounding factors such as sex and age.

Based on the aforementioned findings, being aged 20–40 years, experiencing stigma and perceiving oneself as physically unwell all emerged as significant risk factors for anxiety and depression. Moreover, among individuals with MDR/RR-TB, being male, having faith in recovery and rarely or never experiencing feelings of discrimination or avoidance can potentially mitigate the risk of anxiety and depression.

According to our research, anxiety and depression affected more than half of the patients receiving treatment for MDR/RR-TB, and this emotional burden intensified during the COVID-19 epidemic. Our findings align with the results of face-to-face interviews, which highlighted that respondents' anxiety or depression stemmed from feelings of having an untreatable illness, financial strain, embarrassment regarding their condition and the fear of discrimination from others. The importance of psychological evaluation for patients with MDR/RR-TB is underscored by our findings.

## Discussion

It has been widely reported that patients with chronic and infectious diseases are more likely to exhibit symptoms of anxiety and depression (24, 25), particularly those with pulmonary TB (12, 26–28). Patients diagnosed with MDR/RR-TB are reported to be three times more susceptible to experiencing negative emotions, such as anxiety and despair, largely due to the prolonged treatment duration and high cost of therapy (29). Given the increasing prevalence of MDR/RR-TB, the actual extent of depression and anxiety among patients undergoing treatment is likely to follow an “iceberg phenomenon”. Unfortunately, in clinical practice, the psychological changes and needs of patients are often overlooked, which can lead to adverse outcomes, such as treatment discontinuation or loss (30). Furthermore, the COVID-19 pandemic has heightened the risk of anxiety and depression among patients with MDR/RR-TB given the additional stressors in their daily lives (31). In light of these new challenges, it is imperative to reassess the traumatic experiences of patients with MDR/RR-TB; however, the existing research remains inadequate. To address this gap and determine the prevalence of anxiety and depression among patients with MDR/RR-TB amidst the COVID-19 pandemic, we administered structured questionnaires and conducted in-person interviews with patients undergoing treatment at Guangzhou Chest Hospital.

Upon further evaluation of the patients' GAD-7 and PHQ-9 scores, the incidence of anxiety among patients with MDR/RR-TB was found to be 57.53%, surpassing rates reported in China (32) and Pakistan (33) (29.34 and 47.2%, respectively). The incidence of depressive states was 65.75%, exceeding rates reported in China (32), Ethiopia (34) and Nigeria (35) [29.94%, 51.9% (95% CI: 42.7–62.2%) and 45.5% (*P* < 0.001), respectively], but consistent with the rate in Cameroon (61.1%, 95% CI: 55.1–66.8) (36). The combined prevalence of anxiety and depression was 33.3% (*n* = 73), higher than that reported in a study conducted in Liaoning, northeast China (28) (*n* = 160, 12.78%). Our results indicate that patients with MDR/RR-TB in Guangzhou experience more severe anxiety and depression than those in similar trials conducted in other regions, despite variations in the frequency of these conditions between studies, potentially attributed to variations in research area, nationality and population demographics. Furthermore, this study included both outpatients and inpatients, unlike previous studies that predominantly focused on mental health disorders in inpatients and overlooked outpatients, potentially contributing to the higher prevalence noted above. It is worth noting that we assessed the self-reported frequency and severity of anxiety and depression in the MDR/RR-TB population during the previous 2 weeks, rather than anxiety and depression diagnoses by psychiatrists. Short-term anxiety and depressive states are prevalent in patients (37), which may further explain the elevated prevalence observed in our study. The emotional wellbeing of patients with MDR/RR-TB demands urgent attention from healthcare professionals to prevent the exacerbation of negative psychological outcomes.

### Stigma is one of the factors that leads to psychological illnesses

We identified stigma as the primary factor contributing to anxiety and depression in patients with MDR/RR-TB. In a previous study, patients with MDR/RR-TB were more likely to experience anxiety, depression, tuberculosis-related stigma and low self-affirmation than those with drug-sensitive tuberculosis (32). The harmful adverse effects of anti-TB medications and the stigma associated with TB constitute the main drivers of this stigma. According to a systematic review, most patients experience stigma (38), which can manifest as stress, sadness or anxiety. Additionally, stigma contributes to treatment failure in patients with TB, exerting a severe impact by fostering feelings of melancholy, self-hatred and disillusionment, impeding patients' access to necessary medical care (30). According to previous studies, patients with MDR-TB in some low-income communities conceal their illness due to concerns about discrimination (39), thereby increasing transmission. Therefore, it is imperative to disseminate accurate information about the disease and reassure patients that MDR-TB can be effectively cured with appropriate therapy.

### Lower subjective assessments of physical health

Furthermore, our observations revealed that the majority of patients had lower subjective assessments of their physical health. Drug-resistant TB is a devastating ailment. Infection with *M. tuberculosis* can lead to emaciation or malnutrition, which in turn, compromises patients' physical wellbeing. Combined with the prolonged treatment duration and adverse effects of medication, this deteriorating physical condition exacerbates anxiety and depression. Thus, it becomes imperative to devise individualized, short-term treatment plans and nutritional supplement therapies tailored to the patient's physical condition. Adjustments in medication are necessary to mitigate the adverse effects of drugs and enhance the patient's physical resilience.

### Lack of self-efficacy

Self-efficacy is another crucial element in the association between anxiety and depression. Self-efficacy is the conviction that one can successfully plan and conduct activities (40). Patients with high self-efficacy tend to have more optimistic views of their potential to recover from sickness and achieve a better quality of life (41), which helps them complete their treatment. Given the lack of knowledge about the illness, the majority of patients are afraid of it and perceive it as incurable, especially patients with MDR/RR-TB, who require longer treatment times than drug-sensitive patients and may even have recurring episodes. Therefore, social support from doctors and families becomes imperative for improving self-efficacy and maintaining optimism.

### Female patients need to be given more emotional support

Disparities were uncovered in the association between sex and anxiety and depression, with males exhibiting a more protective stance against anxiety onset compared to females. Females tended to experience more severe anxiety, particularly at moderate levels and above. A study conducted in China indicated that women face a 1.75-fold higher risk of depression compared to men (42). Interestingly, we did not observe this correlation in our study, although women's PHQ-9 scores remained higher than those of men, potentially attributable to variations in the depression assessment tools employed across different studies. Women are inherently at a greater risk of experiencing mental disorders (42), including anxiety and depression. Given their heightened emotional sensitivity, women may perceive greater stigma when grappling with such conditions (43). Moreover, their social participation and support networks may be more limited than those of men, rendering them less adaptable to the same illnesses. Despite female patients constituting only one-third of the MDR/RR-TB cohort, our findings indicate that their anxiety and depression levels surpass those of male patients. Given the limited research specifically examining sex differences, it is imperative to devote greater attention to the mental wellbeing of female patients.

### The bitterness of middle-aged patients with MDR/RR-TB

Similar to other studies (28, 34), we demonstrated that patients aged 20–40 years with MDR/RR-TB were at greater risk of anxiety and depression. This age cohort typically constitutes the primary social workforce, and amidst the economic downturn catalyzed by COVID-19, the imperative for earning a livelihood has intensified. Consequently, anxiety and depression have worsened due to multifaceted stressors stemming from familial, financial and treatment-related concerns. Moreover, income level emerged as a social determinant influencing the likelihood of anxiety occurrence. Although our study did not establish a direct correlation between income and psychological disorders, face-to-face interviews revealed that individuals with lower income levels often grapple with financial anxieties, particularly those with MDR/RR-TB requiring prolonged treatment and encountering substantial medical expenses, sometimes reaching catastrophic levels (44). The COVID-19 pandemic has further compounded economic pressures, amplifying the incidence of anxiety and depression. To alleviate the financial and psychological strain on patients and avert treatment interruptions stemming from the inability to afford care, health policymakers must devise more robust supportive measures tailored to the treatment of patients with MDR/RR-TB.

## Conclusions

Patients with MDR/RR-TB are experiencing heightened levels of depression and anxiety amidst the backdrop of the COVID-19 pandemic, potentially attributable to various factors such as disease stigma, self-health efficacy and socioeconomic conditions. The forthcoming priority in comprehensive care for MDR/RR-TB revolves around ensuring the mental wellbeing of patients with MDR/RR-TB throughout their treatment journey, a critical endeavor for bolstering the quality of medical services and fostering patients' overall health and wellbeing. For instance, primary healthcare facilities should implement full-cycle treatment support services that place equal emphasis on patients' physical and mental health. Routine assessments of patients' depression and anxiety levels before, during and after treatment should be conducted, with psychological support provided by volunteers from non-governmental organizations and psychological clinics. Health education initiatives covering topics such as drug-resistant tuberculosis and patient care techniques must be extended to the families of patients, who should be encouraged to offer immediate emotional assistance. Moreover, we advocate for the implementation of more viable financial strategies by health authorities to mitigate adverse outcomes, such as treatment discontinuation resulting from unmanageable costs.

## Data availability statement

The datasets presented in this article are not readily available because data cannot be shared publicly due to privacy protections. Researchers who meet the criteria for access to confidential data can obtain data from the Ethics Committee of Guangzhou Chest Hospital. Requests to access the datasets should be directed to XX-c, gzcdc_xiaoxc@gz.gov.cn.

## Author contributions

ZD-n: Data curation, Investigation, Writing – original draft, Writing – review & editing. ZG-m: Data curation, Formal analysis, Investigation, Writing – original draft. DY-h: Methodology, Resources, Writing – review & editing. LY: Project administration, Software, Writing – review & editing. WT: Formal analysis, Methodology, Software, Writing – review & editing. CY-y: Investigation, Writing – review & editing. XY-h: Investigation, Writing – review & editing. XX-c: Conceptualization, Writing – review & editing, Funding acquisition, Resources, Supervision.
